# *Kctd7* deficiency induces myoclonic seizures associated with Purkinje cell death and microvascular defects

**DOI:** 10.1242/dmm.049642

**Published:** 2022-09-13

**Authors:** Justine H. Liang, Jonathan Alevy, Viktor Akhanov, Ryan Seo, Cory A. Massey, Danye Jiang, Joy Zhou, Roy V. Sillitoe, Jeffrey L. Noebels, Melanie A. Samuel

**Affiliations:** 1 Department of Neuroscience, Baylor College of Medicine, Houston, TX 77030, USA; 2 Huffington Center on Aging, Baylor College of Medicine, Houston, TX 77030, USA; 3 Developmental Neurogenetics Laboratory, Department of Neurology, Baylor College of Medicine, Houston, TX 77030, USA; 4 Department of Human and Molecular Genetics, Baylor College of Medicine, Houston, TX 77030, USA; 5 Department of Pathology and Immunology, Baylor College of Medicine, Houston, TX 77030, USA; 6 Jan and Dan Duncan Neurological Research Institute of Texas Children's Hospital, Houston, TX 77030, USA

**Keywords:** *Kctd7*, Epilepsy, Seizure, Motor defects, Cerebellum, Vasculature

## Abstract

Mutations in the potassium channel tetramerization domain-containing 7 (*KCTD7*) gene are associated with a severe neurodegenerative phenotype characterized by childhood onset of progressive and intractable myoclonic seizures accompanied by developmental regression. *KCTD7*-driven disease is part of a large family of progressive myoclonic epilepsy syndromes displaying a broad spectrum of clinical severity. Animal models of *KCTD7*-related disease are lacking, and little is known regarding how KCTD7 protein defects lead to epilepsy and cognitive dysfunction. We characterized *Kctd7* expression patterns in the mouse brain during development and show that it is selectively enriched in specific regions as the brain matures. We further demonstrate that *Kctd7*-deficient mice develop seizures and locomotor defects with features similar to those observed in human *KCTD7*-associated diseases. We also show that *Kctd7* is required for Purkinje cell survival in the cerebellum and that selective degeneration of these neurons is accompanied by defects in cerebellar microvascular organization and patterning. Taken together, these results define a new model for *KCTD7*-associated epilepsy and identify *Kctd7* as a modulator of neuron survival and excitability linked to microvascular alterations in vulnerable regions.

## Abstract (precis)

**Summary:** *Kctd7* knockout mice develop seizures and locomotor defects, mirroring *KCTD7*-driven progressive myoclonic epilepsy in humans, and show neuronal cell death in the cerebellum and region-specific defects in brain microvasculature.

## INTRODUCTION

Mutations in the potassium channel tetramerization domain-containing (KCTD) 7 (*KCTD7*) gene cause progressive myoclonic epilepsy (PME) 3 (OMIM 611726), a rare but often crippling neurodevelopmental and epileptic disorder. *KCTD7*-related progressive myoclonic epilepsy (*KCTD7*-PME) has been reported in over 55 patients with more than 40 unique variants (Burke et al., 2021; Dai et al., 2019; Dudipala et al., 2021; Farhan et al., 2014; Kousi et al., 2012; Kozina et al., 2020; Lindy et al., 2018; Mastrangelo et al., 2019; Mei et al., 2019; Metz et al., 2018; Rahman and Fatema, 2021; Vairo et al., 2017; Van Bogaert et al., 2007; Blumkin et al., 2012; Moen et al., 2016). Affected patients carry homozygous or compound heterozygous mutations, whereas heterozygous family members are neurologically unaffected (Metz et al., 2018). Patients with PME are characterized by early-onset seizures, delayed development, ataxia, motor control defects and, in some cases, progressive microcephaly (Van Bogaert, 2016). Some *KCTD7*-PME patients also display features consistent with neuronal ceroid lipofuscinosis [or ceroid lipofuscinosis, neuronal (CLN)] characterized by the accumulation of intracellular autofluorescent lipid material (Mastrangelo et al., 2019; Staropoli et al., 2012). The severe disease characteristics of *KCTD7*-PME are consistent with the widespread expression of KCTD7 throughout multiple brain areas in adults (Kousi et al., 2012; Azizieh et al., 2011).

Since the first reports of *KCTD7*-associated PME appeared over a decade ago, there has been a growing interest in KCTD biology. KCTD7 belongs to a family of 25 potassium channel tetramerization domain proteins with largely unknown cellular function. All members contain the T1 tetramerization domain of voltage-gated potassium channels (Kreusch et al., 1998; Stogios et al., 2005), and KCTD7 has been variably associated with neuronal potassium channel function (Azizieh et al., 2011 (Metz et al., 2018; Staropoli et al., 2012; Liu et al., 2013), tumorigenesis (Angrisani et al., 2021) and autophagy (Metz et al., 2018). Even less is known about the precise molecular and cellular biology underlying *KCTD7*-driven human pathology, and no mouse models of the neurological disease have as yet been reported, apart from our recent study of *Kctd7*-linked retinal function and vascular defects (Alevy et al., 2019). As anti-epileptic treatments are only partially effective in mitigating *KCTD7*-dependent seizure activity or attenuating the course of other neurological defects, there is a critical need to model and understand the pathophysiology of *KCTD7-*associated disease.

Toward this goal, we first characterized *Kctd7* expression patterns in the developing mouse brain. We found that *Kctd7* expression emerges early in postnatal development and is further enriched in specific brain regions, including the cerebellum, hippocampus and olfactory bulb, as the brain matures. We then examined the cortical excitability phenotype of mice lacking both copies of the *Kctd7* gene and discovered that young mutants display many of the signature features of human *KCTD7*-PME, including juvenile electrographic and behavioral myoclonic seizure activity and impaired motor function, accompanied by poor survival of Purkinje neurons and defects in the cerebellar microvasculature. We conclude that *Kctd7* is indispensable for the proper development and maintenance of neuron network activity, which, in turn, is correlated with reduced Purkinje neuron survival and abnormal cerebellar microvascular organization. These results provide a model for KCTD-associated PME that may be useful for understanding human disease progression and mitigating pathology.

## RESULTS

### *Kctd7* expression is developmentally regulated

**Fig. 1.**
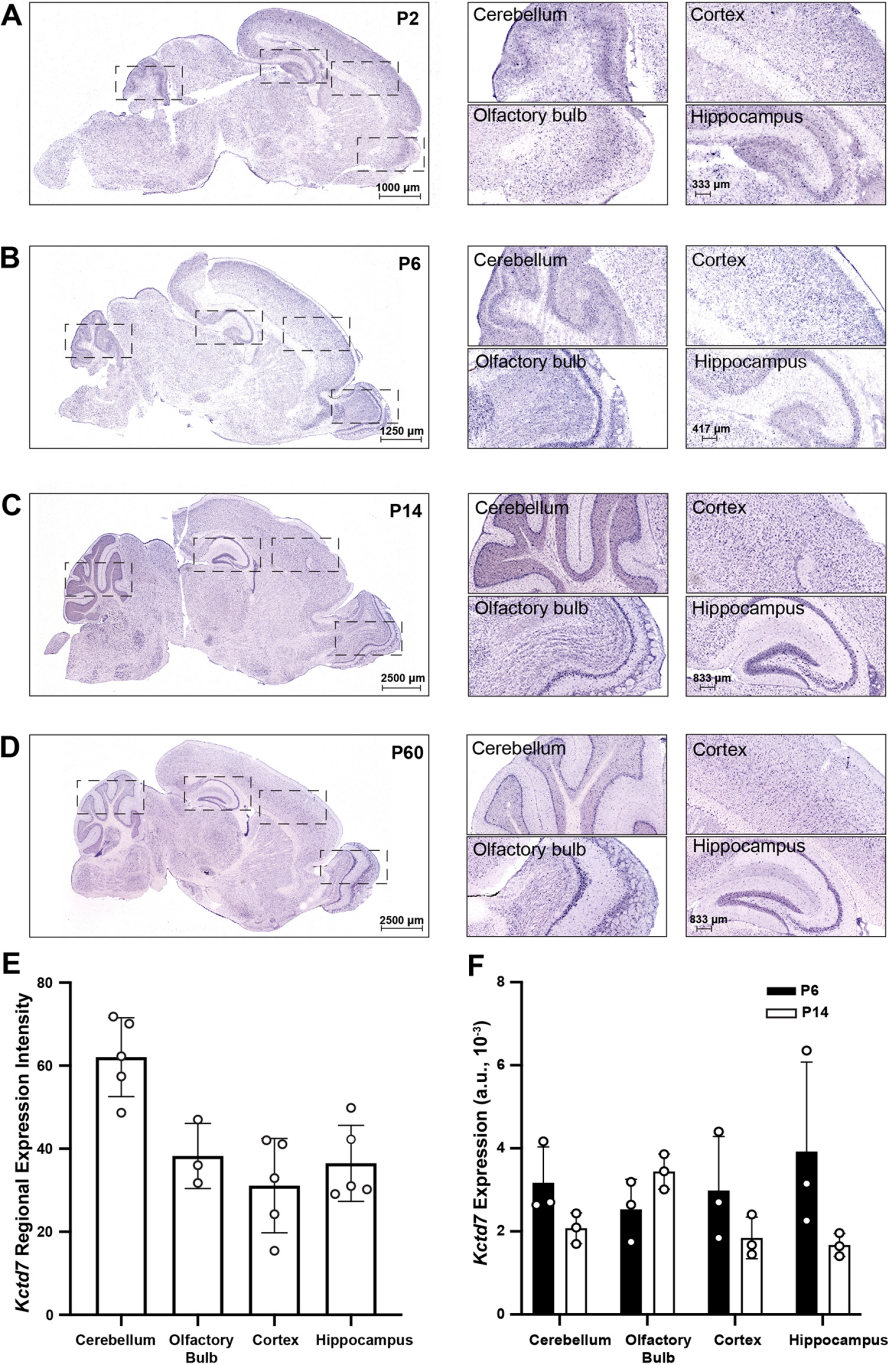
***Kctd7* expression is developmentally regulated.** (A-D) The spatiotemporal localization of *Kctd7* mRNA was examined in wild-type animals by *in situ* analysis for *Kctd7* at four developmental ages. Boxed areas (left) are shown at higher magnification (right). At P2 (A) and P6 (B), *Kctd7* transcripts were present at low levels throughout the brain with modest enrichment within the actively developing cerebellum and olfactory bulb. By P14 (C), *Kctd7* expression levels increased markedly, with particular enrichment within the cerebellar cortex, hippocampal formation and olfactory bulb. In adults (D), *Kctd7* expression was maintained at high levels, as was region specificity, with a laminar gradient present in the neocortex and higher levels in the cerebellar Purkinje cell layer, hippocampal formation and olfactory bulb. Images are representative of *n*=3 animals. (E) Quantification of *Kctd7 in situ* expression patterns in adulthood. Data are presented as the relative signal intensity in a given brain region sampled sagittally across the brain over three to five *in situ* optical sections. (F) Brains from wild-type mice (*n*=3 per time point) were analyzed at P6 and P14 for *Kctd7* mRNA by qRT-PCR. Values represent the fold mRNA expression level relative to *Gapdh*. Data are represented as the mean±s.e.m. a.u., arbitrary units.

*KCTD7*-driven PME is characterized by early disease onset (Van Bogaert, 2016). As reliable antibodies against Kctd7 for immunohistochemistry are not available, we used *in situ* hybridization for *Kctd7* mRNA to examine the timing and specificity with which *Kctd*7 gene expression patterns emerge in sagittal brain sections of wild-type animals during postnatal development. We focused our *in situ* analyses on key brain maturation time points, postnatal day (P) 2, P6, P14 and P60 (Fig. 1A-E). We found that *Kctd7* mRNA transcripts were enriched in the developing cerebellum and hippocampus as early as P2-P6, and by P14 showed intense expression in cerebellar, hippocampal, and olfactory bulb neurons. This enrichment was maintained into adulthood (Fig. 1A-D). To confirm and extend these results, we quantified and compared *Kctd7* levels at P60 to determine how expression levels vary across regions. We found a 1.5-fold enrichment of *Kctd7* signal in the P60 cerebellum relative to the forebrain (Fig. 1E), suggesting that this region might be a key site of Kctd7 activity to prevent ataxic motor deficits in this disorder. We also identified intense *Kctd7* expression at this time point in the entorhinal cortex and the subthalamic nucleus, regions linked to epilepsy and focal motor phenotypes (Ren et al., 2020) (Fig. S1). In parallel, we performed quantitative real-time PCR (qRT-PCR) to measure *Kctd7* levels in the hippocampus, brain stem, cerebellum, cortex and olfactory bulb at P6 and P14 (Fig. 1F). The relative gene expression levels were comparable to the *in situ* hybridization patterns and intensities at these time points (Fig. 1B,C). These data indicate that *Kctd7* expression is temporally regulated, attains high levels during early postnatal time points, and is extensively maintained into adulthood.

### *Kctd7*-deficient mice display myoclonic seizures and locomotor defects that share features of human disease

**Fig. 2.**
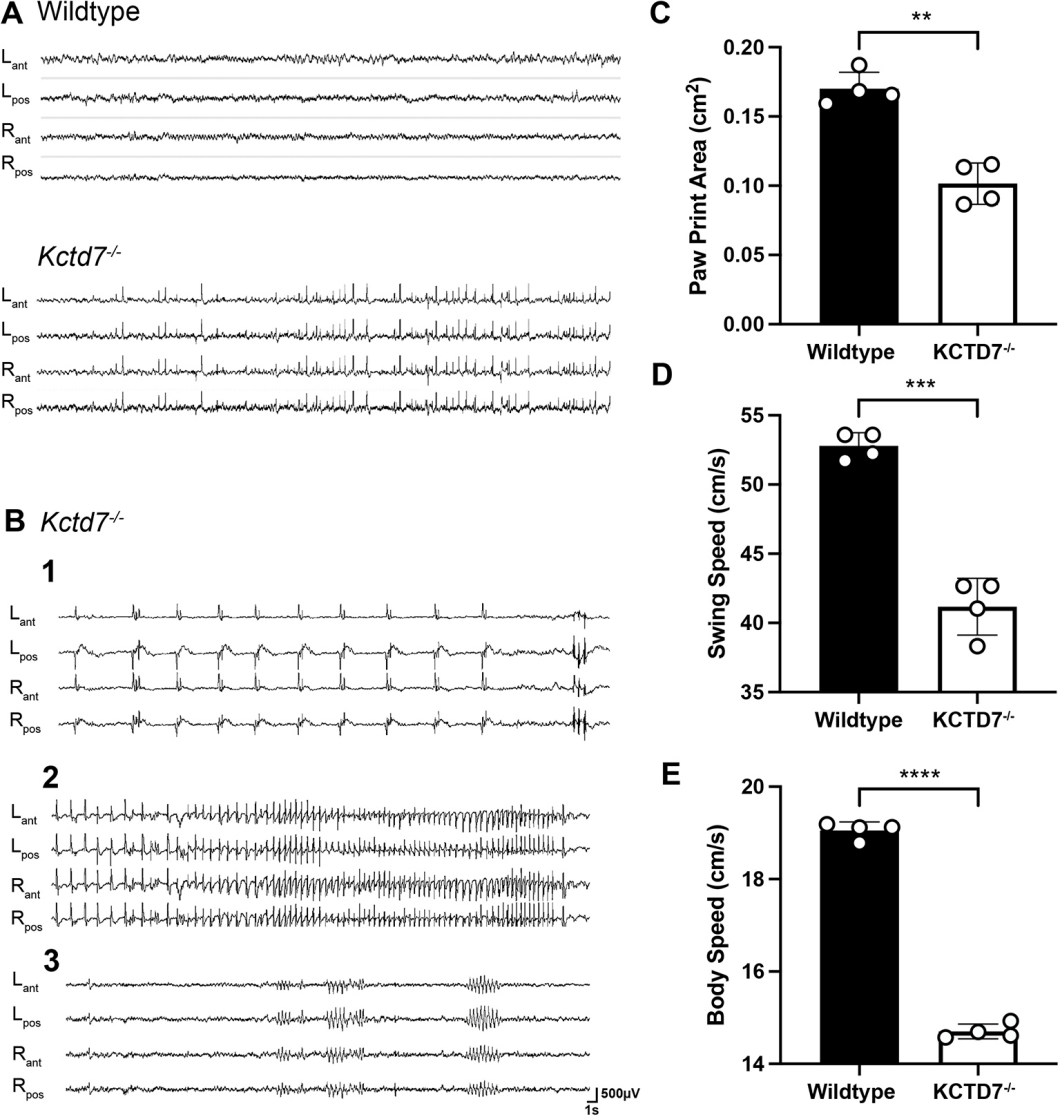
***Kctd7*-deficient mice display robust epileptiform activity and locomotor deficits.** (A,B) Representative video EEG monitoring of awake and behaving wild-type and 2-month-old *Kctd7*^−/−^ mice during interictal (A) and ictal (B) periods. In A, *Kctd7*^−/−^ mice showed pronounced bilateral cortical fast-spike and polyspike discharges that were not detected in wild-type controls. In B, recordings from three different animals are shown. (B-1) Isolated bilateral spike and spike-wave complexes were each accompanied by distinct myoclonic jerks of the head. (B-2) Generalized seizure discharges were accompanied by sustained myoclonic activity and could last from several seconds to 1-2 min. (B-3) Brief episodes of 6 s spike-wave discharges with behavioral arrest were also detected. Electrode locations: L_ant_, left anterior; L_pos_, left posterior; R_ant_, right anterior; R_pos_, right posterior. (C-E) Spontaneous gait CatWalk analysis was performed on 2-month-old wild-type (*n*=9 animals) and *Kctd7*^−/−^ mice (*n*=12 animals) mice to analyze gross motor coordination, stance and stride patterns. *Kctd7^−/−^* mice displayed significant decreases in footprint area (cm^2^) (C), swing speed (cm/s) (D) and body speed (cm/s) (E) for all four paws. Data are represented as a comparison of averaged values across all four paws between control (*n*=9) and mutant (*n*=12) mice. Data are represented as the mean±s.e.m. **P<0.003; ***P<0.0003; ****P<0.0001; unpaired two-tailed Welch's *t*-test for significance.

**Fig. 3.**
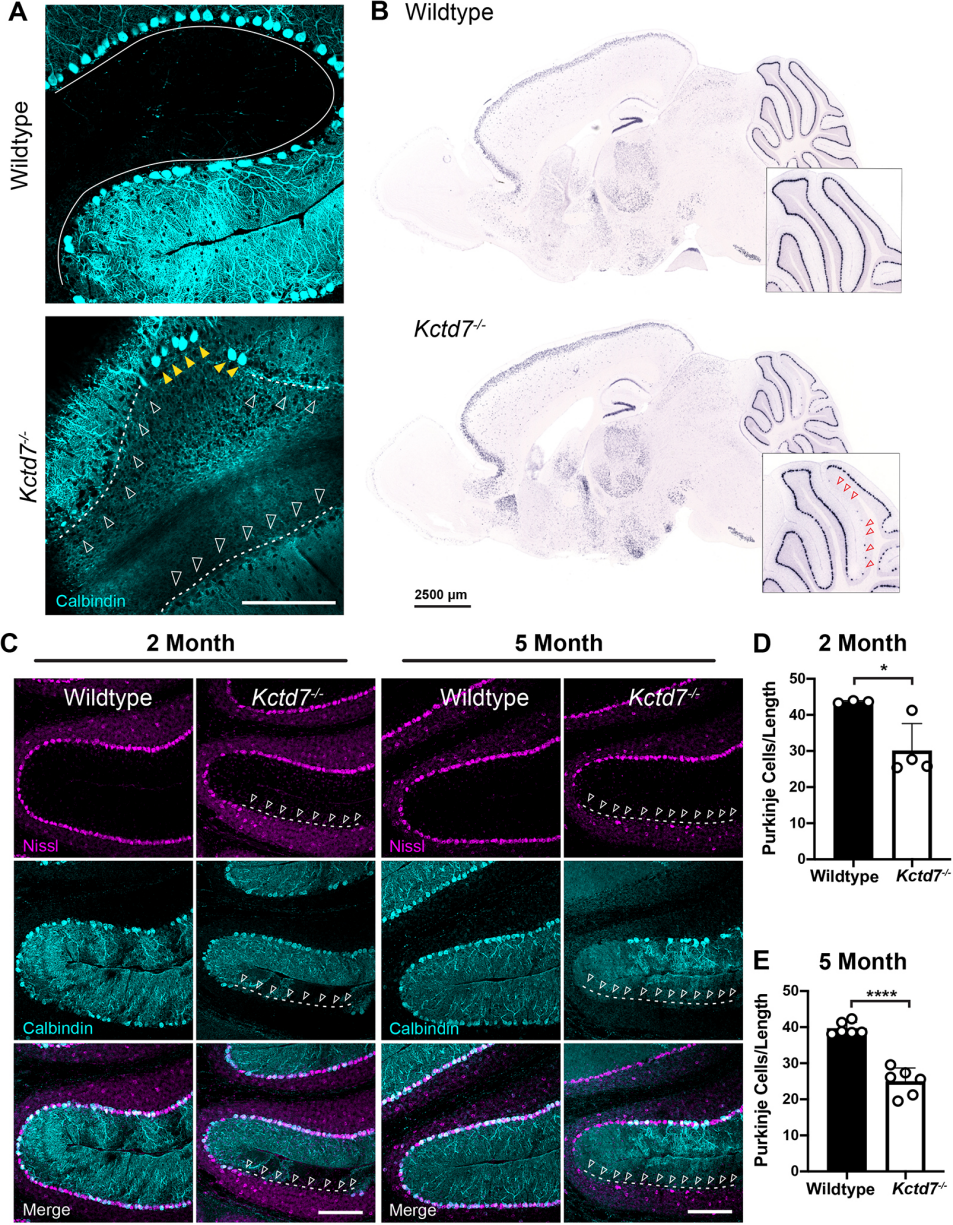
***Kctd7* is required for Purkinje neuron survival.** (A,B) The numbers and localization of calbindin-positive Purkinje neurons were assayed by immunohistochemistry analysis (A) and *in situ* hybridization (B) for calbindin in adult 2-month-old mice. In wild-type animals, Purkinje neurons form a single, continuous layer that clearly traces each cerebellar lobule (solid line). In *Kctd7*^−/−^ mice, clear loss of Purkinje neurons is apparent in both visualization methods, as indicated by large gaps in the calbindin-positive layer [indicated by dashed lines and unfilled white (A) and red (B) arrowheads]. Yellow arrowheads in A indicate remaining Purkinje neurons in the *Kctd7* mutant. (C-E) The distribution (C) and number (D,E) of Purkinje neurons were quantified at 2 and 5 months of age in wild-type controls (*n*=3 and 6 animals for 2 and 5 months, respectively) and mutant animals (*n*=4 and 6 animals for 2 and 5 months, respectively). Purkinje neuron numbers were significantly reduced at both time points, indicating that Purkinje neuron loss is an early feature in *Kctd7*^−/−^ mice. Data are represented as the mean±s.e.m. *P<0.05; ****P<0.0001; unpaired two-tailed *t*-test for significance. Scale bars: 200 μm (A,C); 2.5 mm (B).

*KCTD7*-PME patients display myoclonic or generalized tonic-clonic seizures with abnormal electroencephalograms (EEGs) featuring frequent multifocal and/or generalized spike waves associated with an excess of slow activity (Dai et al., 2019; Dudipala et al., 2021; Farhan et al., 2014; Kousi et al., 2012; Mastrangelo et al., 2019; Mei et al., 2019; Van Bogaert et al., 2007; Blumkin et al., 2012; Moen et al., 2016; Staropoli et al., 2012; Krabichler et al., 2012). We performed chronic video-EEG monitoring in 2-month-old moving young adult *Kctd7*-deficient mice and detected robust spontaneous epileptiform activity, consisting of interictal multifocal fast cortical spike and polyspike discharges in eight of ten animals recorded (Fig. 2A). In seven of these mice, the spikes triggered clearly observable episodes of single myoclonic jerks of the head, with a sudden head/shoulder drop (Fig. 2B-1). Behavioral myoclonic seizures were also noted (Fig. 2B-2), consisting of high-frequency runs of repetitive spike discharges with a myoclonic head drop and clonic truncal and limb movements, followed in some instances by abnormal tremor and repetitive grooming and movements of the forelimbs (Movie 1). These episodes could last from several seconds to 1-2 min. Occasional brief episodes of 6 s spike-wave discharges with behavioral arrest were also detected (Fig. 2B-3). No generalized tonic-clonic convulsions were observed, and all seizure events were immediately followed by a return to normal EEG activity and motor behavior upon termination of the seizure.

Human *KCTD7* patients display pronounced motor defects that can manifest as ataxia, tremors and dyskinesia (Dai et al., 2019; Farhan et al., 2014; Kousi et al., 2012; Kozina et al., 2020; Mastrangelo et al., 2019; Blumkin et al., 2012; Staropoli et al., 2012). As *Kctd7* is heavily expressed in the cerebellum and enriched in Purkinje neurons, which regulate and coordinate motor movements (Fig. 1; Sathyanesan et al., 2019; Chambers and Sprague, 1955; Morton and Bastian, 2007; Valle et al., 2017), we tested whether seizures in *Kctd7*-deficient mice were accompanied by interictal motor coordination deficits. We employed the footprint test to evaluate gait, and the accelerating rotarod and parallel grid footslip assays to evaluate limb coordination at 2 months of age. *Kctd7*-deficient mice displayed defects in gait, as the print area, swing speed and body speed were all decreased for each of the four paws (Fig. 2C-E). Despite the gait defect at this age, limb coordination as measured by both the parallel grid footslip assay and accelerating rotarod test appeared normal, with no significant difference in the number of footfalls and periods of immobility (Fig. S2A,B). These early alterations did not continue to progress, as similar findings were observed at 5 months of age (Fig. S2C). Taken together, these data indicate that mice deficient in *Kctd7* develop some features of human disease-associated gait alterations. However, the more extensive ataxia and progressive lack of motor coordination reported in many human cases were not observed in this model.

### Loss of *Kctd7* is accompanied by sporadic Purkinje neuron degeneration

To examine the anatomical basis for the deficits, we carried out a histological analysis of the brain in age-matched control and *Kctd7^−/−^* mice. To evaluate neuronal integrity, we utilized a panel of neuron-specific mRNA and antibody markers (Table 1). We sampled a range of brain regions, with a particular focus on the cerebellum, given its high levels of *Kctd7* expression and key role in motor coordination. We used an anti-calbindin antibody to mark Purkinje neurons and distinguish them from Bergman glia, and quantified calbindin-positive cells by *in situ* hybridization and immunohistochemistry. We observed a patchy loss of Purkinje neurons in adult *Kctd7^−^*^/−^ mice, with small gaps and larger cell-free stretches in a number of cerebellar folia regions (Fig. 3A,B). To investigate the timing of the cell loss, we focused subsequent analysis on anterior and central lobules V/VI, given their critical involvement in locomotion and ataxia (Chambers and Sprague, 1955; Morton and Bastian, 2007; Valle et al., 2017). In this region, we observed a somewhat progressive loss of Purkinje neurons at 2 months of age, with a 31% reduction (*P*=0.0290, Fig. 3C,D) compared to wild-type mice. By 5 months of age, 37% of the Purkinje neurons were lost (*P*<0.0001, Fig. 3C,E). To confirm that this reduction was not due to a *Kctd7*-dependent decline in calbindin levels in vulnerable cells, we stained cells for both carbonic anhydrase related protein 8 (CAR8, encoded by *Car8*) and inositol 1,4,5-trisphosphate receptor 1 (IP3R1, encoded by *Itpr1*), selective markers of Purkinje neurons. Both markers validated a similar loss of Purkinje neurons in *Kctd7^−/−^* mice (Fig. S3A,B). The requirement for *Kctd7* for neuronal survival within the cerebellum appeared to be specific to Purkinje cells, as the average thickness of the underlying granule cell layer did not differ between controls and mutants (Fig. S4). To test the regional specificity of neuronal loss, we also examined the dentate gyrus and CA1 region of the hippocampus. Staining with a diverse array of neuron subtype-specific markers revealed no apparent change in neuron distribution, organization or number in these regions (Fig. S5). Collectively, these data suggest that *Kctd7* is required for long-term survival of Purkinje neuron subsets, consistent with the early and progressive motor deficits observed in *KCTD7*-associated PME.

**Table 1.**
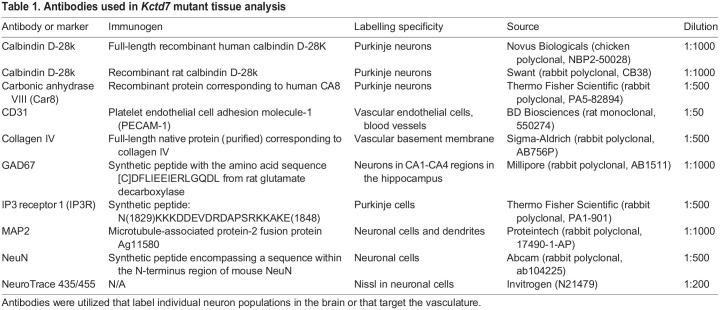
Antibodies used in *Kctd7* mutant tissue analysis

### Cerebellar vasculature defects accompany neuron loss in *Kctd7*-deficient mice

**Fig. 4.**
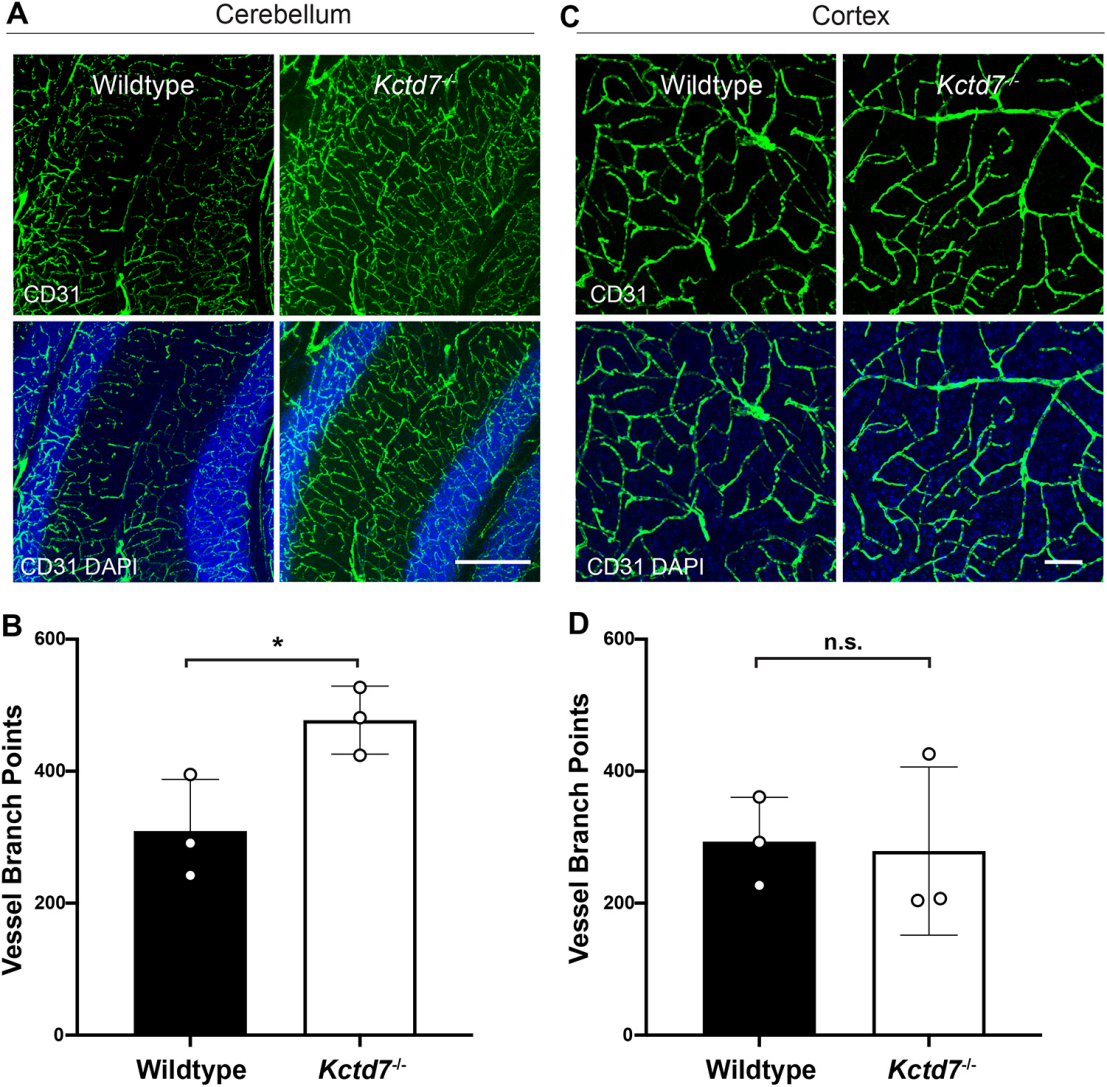
**Cerebellar microvascular defects accompany neuron loss in *Kctd7^−/−^* mice.** To examine brain microvascularization patterns, 2-month-old wild-type control and *Kctd7*^−/−^ brains were collected, sectioned and stained with antibodies against CD31. (A,C) Representative images of the cerebellar (A) and cortical (C) microvascular plexus are shown. (B,D) Differences in vessel branching were quantified in wild-type controls and *Kctd7*^−/−^ mice by counting the number of vascular branch points in the cerebellum (B) and cortex (D). Significant increases in vascular branching were observed in the cerebellum but not in the cortex of *Kctd7*^−/−^ mice. *n*=3 wild-type and *n*=3 *Kctd7*^−/−^ animals. Scale bars: 200 µm (A); 100 µm (C). Data are represented as the mean±s.e.m. n.s., not significant; *P<0.05; unpaired two-tailed *t*-test for significance.

Using the same *Kctd7^−/−^* mouse model, we previously showed that *Kctd7* is required for neurovascular development and patterning in the retina (Alevy et al., 2019). We questioned whether these defects might extend to the brain. Changes in the vasculature and underlying regulatory pathways, as mediated by vascular endothelial growth factor (VEGF) have been increasingly observed in human and experimental models of epilepsy (Croll et al., 2004; Morin-Brureau et al., 2011; Ndode-Ekane et al., 2010; Sun et al., 2016; Vezzani, 2005; Zhai et al., 2016; Tang et al., 2017). In the cerebellar cortex, Purkinje cells are highly vulnerable to late-onset degeneration and could be sensitive to abnormal neurovascular coupling, which requires a properly patterned microvasculature (Mapelli et al., 2017). We therefore began our investigation by examining the cerebral capillary network at 2 months of age using an antibody against CD31 (also known as platelet endothelial cell adhesion molecule 1 or PECAM-1) (Muller et al., 1989). The microvascular network within the cerebellum of *Kctd7*-deficient mice showed an increase in vessel branching relative to age-matched controls (54% increase, *P*=0.0359, Fig. 4A,B). These alterations might be specific to regions in which high levels of *Kctd7* are present, as vessel organization and branching were unaltered in the neocortex relative to controls (*P*=0.8714, Fig. 4C,D). These results indicate that *Kctd7* is required for maintaining proper regional organization of the brain microvasculature and that Purkinje cell loss in the cerebellum is accompanied by alterations in local microvascular organization.

## DISCUSSION

Since the identification of *KCTD7* as an autosomal recessive, progressive myoclonus epilepsy-associated gene over 15 years ago, relatively little progress has been made toward understanding the pathophysiology linking *KCTD7* to human neurodevelopmental disease. Here, we identify key cellular and behavioral phenotypes in a *Kctd7^−/−^* mouse line that recapitulate critical features of the human disorder. In wild-type animals, we find that although low levels of *Kctd7* are broadly expressed, *Kctd7* is enriched in specific forebrain and cerebellar areas during postnatal development and is maintained at high levels into adulthood. *Kctd7^−/−^* mice displayed functional deficits corresponding to the regions in which *Kctd7* is normally enriched (e.g. neocortex, hippocampus and the cerebellar Purkinje cell layer) that might contribute to several key features of human *KCTD7*-associated disease. First, the null mutants developed frequent interictal myoclonic jerks and behavioral myoclonic seizures, including myoclonic jerks of the head and sudden head/shoulder drop. Second, there were early deficits in gait accompanied by segmental loss of cerebellar Purkinje neurons. Although the cell loss and motor deficits were relatively mild in this particular genetic strain, they are consistent with the wider spectrum of cerebellar atrophy and motor movement defects noted in many *KCTD7*-PME patients (Dai et al., 2019; Farhan et al., 2014; Kousi et al., 2012; Kozina et al., 2020; Mastrangelo et al., 2019; Blumkin et al., 2012; Staropoli et al., 2012). Finally, as we found in the developing retina, loss of *Kctd7* resulted in a hitherto unsuspected dysgenesis of cerebellar microvasculature. Together, these data indicate that *Kctd7* function is required to prevent seizures and deficits in motor control, and add *KCTD7-*PME to the increasing number of gene defects linked to disordered brain microvasculature in epilepsy.

Our spatiotemporal expression profiling in mice reveals that although low levels of *Kctd7* are broadly expressed, the gene becomes particularly enriched in the CA1-CA4 regions of the hippocampus, the dentate gyrus granule cell layer, mitral cell layer of the olfactory bulb and Purkinje neurons of the cerebellum as development proceeds. These data are consistent with previous reports (Kousi et al., 2012; Azizieh et al., 2011). Although additional studies are needed, a low-resolution survey of cortical and hippocampal morphology and neuron density did not reveal *Kctd7*-dependent alterations. In contrast, although *Kctd7* is uniformly expressed in all Purkinje cells, some appear more dependent on *Kctd7* for their survival, albeit in a segmental manner that crosses the boundaries of known cerebellar cortex compartments (Sarna and Hawkes, 2003). These data are consistent with early motor function declines reported in the majority of *KCTD7* human patients (Van Bogaert, 2016; Teng et al., 2019) as well as more rarely reported cerebellar atrophy (Moen et al., 2016; Staropoli et al., 2012).

Several possible mechanisms can be explored to account for the Purkinje cell loss. Purkinje cells display sporadic susceptibility to diseases that alter protein function, aggregation and homeostasis, or lysosome function. These diseases include the large family of spinocerebellar ataxias (SCAs), which involve microsatellite repeat expansions or point mutations, Huntington's disease and tuberous sclerosis complex-related autism spectrum disorder (Ashizawa et al., 2018; Reith et al., 2013; Rodda, 1981). In addition, their function and survival depend on developmentally regulated wiring outcomes between Purkinje cells, cerebellar granule neurons and other cells in this circuit (van der Heijden and Sillitoe, 2021). Other genes implicated in the early death of these cells include the *Lurcher* allele, a semi-dominant mutation in the δ2 glutamate receptor encoding gene *Grid2* (Armstrong et al., 2011), and *Weaver* (*Girk*2) (Liesi et al., 2000). Although these syndromes alter Purkinje cell survival, only *SCA13* (*KCNC3*) (Hsieh et al., 2020) and *KCTD7* implicate potassium channel function in selective Purkinje cell death. Unlike most SCA genes, *KCTD7* mutations promote an early onset of ataxia.

Potential Kctd7 biological functions include a role in both cellular homeostasis and potassium channel activity, which could alter neuron function. Evidence for Kctd7-dependent cellular homeostasis pathways appears to converge on the lysosome. *KCTD7*-associated disorders have been reported as the lysosomal storage disease CLN type 14 (CLN14). However, these conclusions were drawn primarily from two patients (Mastrangelo et al., 2019; Staropoli et al., 2012), and there is some consensus that *KCTD7*-associated pathologies might be distinct from CLN (Kousi et al., 2012; Metz et al., 2018; Teng et al., 2019). In agreement with this idea, it is possible that CLN-associated findings might be due to the documented interaction of KCTD7 with cullin-3, a component of cullin-RING E3 ubiquitin ligase complexes (Metz et al., 2018; Azizieh et al., 2011). Thus, KCTD7 could modulate proteostasis, and mutations in *KCTD7* could lead to disease through the accumulation of undegraded proteins. However, proteins that are targets of KCTD7-dependent ubiquitination have not been identified. Alternatively (or in addition), Kctd7 might impact potassium conductance. Like other KCTD family proteins, KCTD7 acquired its name from the single N-terminal BTB/POZ (protein bric-a-brac, tramtrak and broad complex/poxvirus zinc finger) domain that bears homology to the T1/BTB voltage-gated potassium channel tetramerization domain required for functional channel assembly (Ji et al., 2016; Skoblov et al., 2013). In support of this idea, expression of *KCTD7* in mouse neurons or *Xenopus* oocytes hyperpolarizes cells, and patient mutations could inhibit potassium flux (Moen et al., 2016; Azizieh et al., 2011). Kctd7 might also directly associate with GABAB receptor subunits and control potassium conductance through G protein-based regulation, as has been documented for KCTD8, KCTD12, KCTD12B and KCTD16 (Fritzius et al., 2017; Schwenk et al., 2010; Seddik et al., 2012). The mechanism underlying *KCTD7* deficiency in regulating seizure activity will require a systematic examination of cellular excitability, beginning within the regions of highest expression.

Although many key disease features were conserved, *Kctd7^−/−^* mice did not display the severity of the locomotor defects nor the progressive nature of the disease described in many reports of human *KCTD7*-associated PME. Whether this reflects differences between mouse and human central nervous system biology, genetic backgrounds or potential differences caused by specific *KCTD7* mutant alleles remains to be determined. We note that PME manifestations in humans also vary widely in their clinical phenotypes and their severity (Teng et al., 2019; Narayanan et al., 2022).

Finally, our results suggest a previously undescribed role for altered cerebellar microvascular patterning in *KCTD7*-associated disease. These results are consistent with our previous work in the murine retina, in which we showed that *Kctd7* deletion altered retinal function and increased vascular branching (Weiner et al., 2019). How might *Kctd7* influence vessel branching? One possibility is that Purkinje neuron loss is causal to the vasculature changes. In the retina, however, vasculature alterations were not accompanied by neuron loss in *Kctd7^−^*^/*−*^ mice, indicating that vessel changes and local cell death can occur independently. An alternative possibility is that defects result from Kctd7-dependent changes in excitability. In line with this idea, voltage-gated potassium channels participate in returning a depolarized cell to a resting state, and Kctd7 might similarly help bring the membrane potential closer to the equilibrium potential after depolarization. Thus, removing Kctd7 might ultimately result in neuron hyperexcitability, which might lead to increased nutrient demand and induce branching alterations. Finally, we cannot exclude the possibility that Kctd7 plays an intrinsic role in the vasculature itself, as potassium current can influence vessel tone and blood flow (Filosa et al., 2006; Iadecola, 2017).

In summary, our findings are the first to establish a clinically relevant model for the study of *KCTD7*-associated human disease with several key conserved features of human pathology. We show that this neuronally enriched protein is required to prevent seizure activity and locomotor defects and that these functional pathologies are accompanied by selective neuronal loss in the cerebellum with microvasculature alterations. Taken together with previous work, these data suggest that Kctd7 plays critical, region-specific roles in nervous system function. These results serve as a starting point to clarify additional aspects of Kctd7 biology in the brain as well as test potential therapeutics for this intractable disease.

## MATERIALS AND METHODS

### Mouse strains

The *Kctd7* mutant mouse (*Kctd7^−/−^*) was provided by the International Mouse Phenotyping Consortium [*Kctd7^em^*^2(*IMPC*)*Bay*^]. The *Kctd7* knockout was generated through a deletion of exon 2, producing a nonfunctional truncated protein through the introduction of a premature stop codon, resulting in a truncated protein fragment that is degraded through nonsense-mediated decay (Fig. S6). Deletion of the expected exon was validated by Sanger sequencing. Animals were maintained on a C57BL/6NJ background, and age-matched, wild-type C57BL/6NJ controls were used for all studies. Female and male mice of approximately equal numbers were included in all studies. Experiments were carried out in accordance with the Guide for the Care and Use of Laboratory Animals of the National Institutes of Health under protocols approved by the Baylor College of Medicine Institutional Animal Care and Use Committee.

### Tissue preparation and immunohistochemistry

Brains were collected from wild-type and/or *Kctd7^−^*^/−^ animals at 2 and 5 months of age. For immunohistochemistry, mice were perfused with PBS followed by 4% paraformaldehyde (w/v). Brains were then fixed overnight in 4% paraformaldehyde/PBS (w/v) and rinsed with additional PBS. Antibody information, dilutions and specificity are detailed in Table 1. The brain was cryoprotected in 30% sucrose overnight, embedded in Optimal Cutting Temperature (OCT) compound (VWR), frozen in methyl butane on dry ice, sectioned into 35 μm slices using a cryostat, and stored in wells filled with PBS. Free-floating sections were incubated in blocking solution [10% normal donkey serum and 0.5% Triton X-100 (v/v)] in PBS for 1 h, followed by incubation with primary antibodies overnight at 4°C and secondary antibodies for 1 h at room temperature. All samples were mounted on Superfrost slides and in Vectashield (Vectorlabs). Images were acquired on an Olympus FluoView FV1200 confocal microscope and processed using Fiji. Images were acquired with the following specifications: Purkinje neuron, vessel and Nissl staining, 1272×1272 µm; hippocampal staining, 634×634 µm.

### Histological quantification

For quantification, images were collected from three to six animals per group with at least three image stacks per animal. For vasculature quantification, brain sections were stained with anti-CD31 antibodies, and the number of branch points were quantified in merged images from 20-50 consecutive optical sections. To examine vessels and neuron subsets and to quantify their numbers and organization, we used antibodies specific for each cell type in a given region (Table 1). The total number of somas for each neuron type was quantified in standardized 1272×1272 µm optical sections from 20-50 consecutive optical sections per image stack. The granular layer thickness was also quantified in standardized 1272 µm×1272 µm optical sections from 20-50 consecutive optical sections per image stack. We report the average of ten length measurements per lobe per animal determined at regions near the vermis.

### qRT-PCR

Brain regions were dissected in ice-cold RNase-free water, and each sample was homogenized separately. Total RNA was purified from each sample using an RNeasy Plus Mini Kit (QIAGEN) according to the manufacturer's instructions. First-strand cDNA synthesis was performed using a complementary DNA synthesis kit (iScript Reverse Transcription Supermix for qRT-PCR; Bio-Rad) according to the manufacturer's protocol. qRT-PCR was performed with primers for *Kctd7* (forward, 5′-CTGCTGCCCCAGGAGTTTCC-3′, and reverse, 5′-GATGAAGTACCGGCCCTCGG-3′) and *Gapdh* (forward, 5′-AGGTCGGTGTGAACGGATTTG-3′, and reverse, 5′-TGTAGACCATGTAGTTGAGGTCA-3′) using iTaq Universal SYBR Green Supermix (Bio-Rad) and a CFX384 Touch Real-Time PCR Detection System (Bio-Rad). Relative quantification was determined using the ΔΔC_t_ method (Livak and Schmittgen, 2001). Genes of interest were normalized to *Gapdh*. Primers were designed in-house using the Primer-BLAST software or obtained from the Harvard Primer Bank and others (Spandidos et al., 2010).

### *In situ* hybridization

*In situ* hybridization was performed by the RNA *In Situ* Hybridization Core at Baylor College of Medicine using an automated robotic platform as previously described (Yaylaoglu et al., 2005). We prepared a digoxigenin (DIG)-labeled riboprobe to *Kctd7* using reverse-transcribed mouse cDNA as a template that was generated from RNA harvested from mouse brain at embryonic day 15 and P7. First-strand cDNA synthesis was performed using the Superscript IV First-Strand Synthesis System (Invitrogen). PCR primers were used to generate cDNA fragments corresponding to the desired riboprobes for *Kctd7* (forward, 5′-GCGATTTAGGTGACACTATAGTTCTGGCTCTGAGCTAAATTCC-3′, and reverse, 5′-GCGTAATACGACTCACTATAGGGCTTTACCCAGCATCTTTCAACC-3′). DIG-labeled riboprobes were synthesized using a DIG RNA labeling kit (Roche) and stored in hybridization buffer at a concentration of 100 ng/µl at −20°C.

For *in situ* hybridization, brains were cryoprotected in 30% sucrose, frozen in OCT, cryosectioned into 20 μm slices, and mounted on Superfrost Plus slides (VWR). Sections were fixed and acetylated before the hybridization procedure, which was performed on a high-throughput platform. The slides were developed chromogenically after a tyramide amplification step (TSA-Plus system, PerkinElmer Life Sciences) using 5-bromo-4-chloro-3-indolyl phosphate (BCIP) and Nitroblue Tetrazolium as a substrate for 20-40 min.

For fluorescence *in situ* hybridization, the samples were developed using tyramide labeled with Cy3 directly (TSA-Plus system) for 15 min and then stained with 4′,6-diamidino-2-phenylindole (DAPI) before mounting in Prolong Diamond (Invitrogen). To quantify fluorescence intensity for *in situ* hybridization (Fig. 1E), region boundaries were manually defined in P60 tissue using the corresponding DAPI images. The relative levels of the signal within each averaged layer were computed, and a background subtraction was applied to remove background noise.

### EEG recording

Silver wire electrodes (0.0008 inch diameter) soldered to a microminiature connector were implanted bilaterally into the subdural space over the frontal and parietal cortex of mice under isoflurane anesthesia (2-4% O_2_). The reference electrode was placed over the right frontal lobe, and the ground electrode was placed over the left frontal lobe. Mice were allowed to recover for several days before simultaneous EEG and behavioral monitoring were performed using a digital video-electroencephalograph (Labchart, ADInstruments) upon mice of either sex while moving freely in the test cage. EEG activity from 13-week-old adult wild-type and mutant genotypes was assayed during multiple separate 24 h recording sessions. EEGs in each mouse were analyzed by two trained observers.

### Behavioral assays

The spontaneous gait of moving mice was analyzed with CatWalk XT systems (Noldus Information) as described by Hamers et al. (2001). On the day of testing, the mice were acclimated for at least 30 min, and then placed on the Catwalk arena and left undisturbed to walk three compliant trials (<25% variation in speed, minimum speed 5 cm/s and maximum duration of 10 s). Print area, swing speed, body speed and stride length were categorized using the Catwalk XT software. For rotarod testing, mice were acclimated to the testing room for at least 30 min and then placed on the rotating rod. The time (latency) of fall from the rotating rod at increasing speeds and continuous acceleration was recorded. The starting speed was 5 rpm, and the rod was accelerated to a final speed of 40 rpm over 300 s. Mice were assayed in four trials per day for 2-4 days. For the parallel rod footslip assay, an apparatus adapted from Kamens et al. (2005) was used to simultaneously assess ataxia and locomotor activity. On the day of testing, the mice were acclimated for at least 30 min to the testing room. Mice were then placed in the chamber and left to freely move for 10 min. The apparatus tracks the position of the mouse (activity time) and the number of footslips. Foot contact at the bottom plate beneath the parallel rod footslip completes a circuit and activates a switch to monitor these features. Locomotion distance, immobile time and mean speed is measured by the ANY-maze tracking software using an overhead camera.

### Statistical analysis

Statistical analyses on neuron numbers and vascular branch number were performed using an unpaired, two-tailed Student's *t*-test. To compare the averaged values of combined paw performances for the CatWalk analysis, we used the unpaired, two-tailed Welch's *t*-test. No statistical analysis was conducted to predetermine sample sizes. Randomization and blinding were not employed. Statistical differences were evaluated using GraphPad Prism 7 software. *P*<0.05 was considered statistically significant.

## Supplementary Material

Supplementary information
