# Supplementary material for: Kctd7 deficiency induces myoclonic seizures associated with Purkinje cell death and microvascular defects
Source: Dis Model Mech. 2022 Sep 13;15(9):dmm049642. doi: 10.1242/dmm.049642 (PMC9509889; doi:10.1242/dmm.049642)
Supplement: Supplementary information [file dmm-15-049642-s1.pdf]

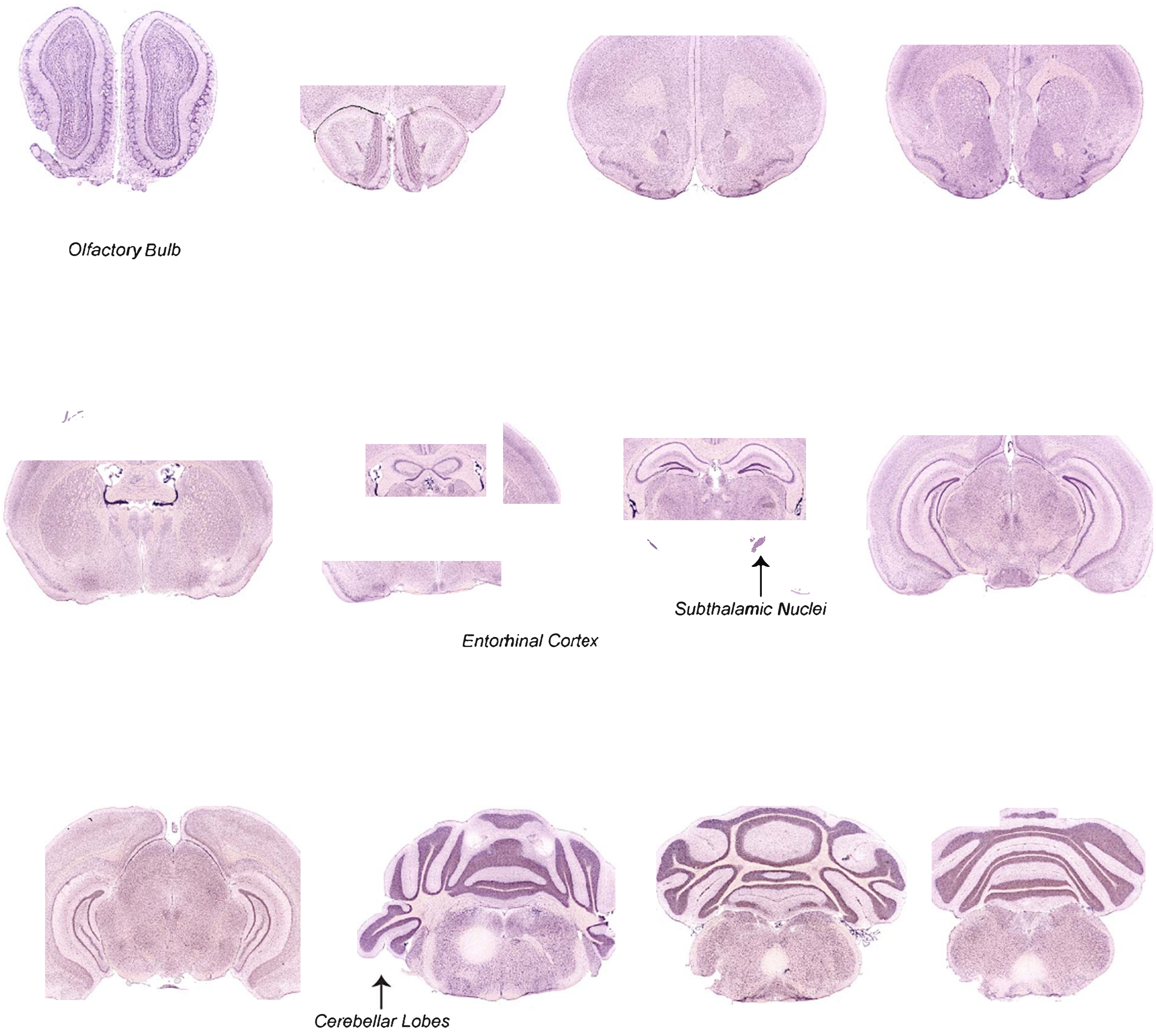


**Fig. S1**. ***Kctd7* expression pattern in coronal brain sections.** The localization of *Kctd7* is shown in coronal sections of wild-type adult mouse brain by *in situ* hybridization. *Kctd7* was notably enriched in the olfactory bulb, hippocampus, cerebellum, entorhinal cortex, and subthalamic nuclei.

Disease Models & Mechanisms • Supplementary information

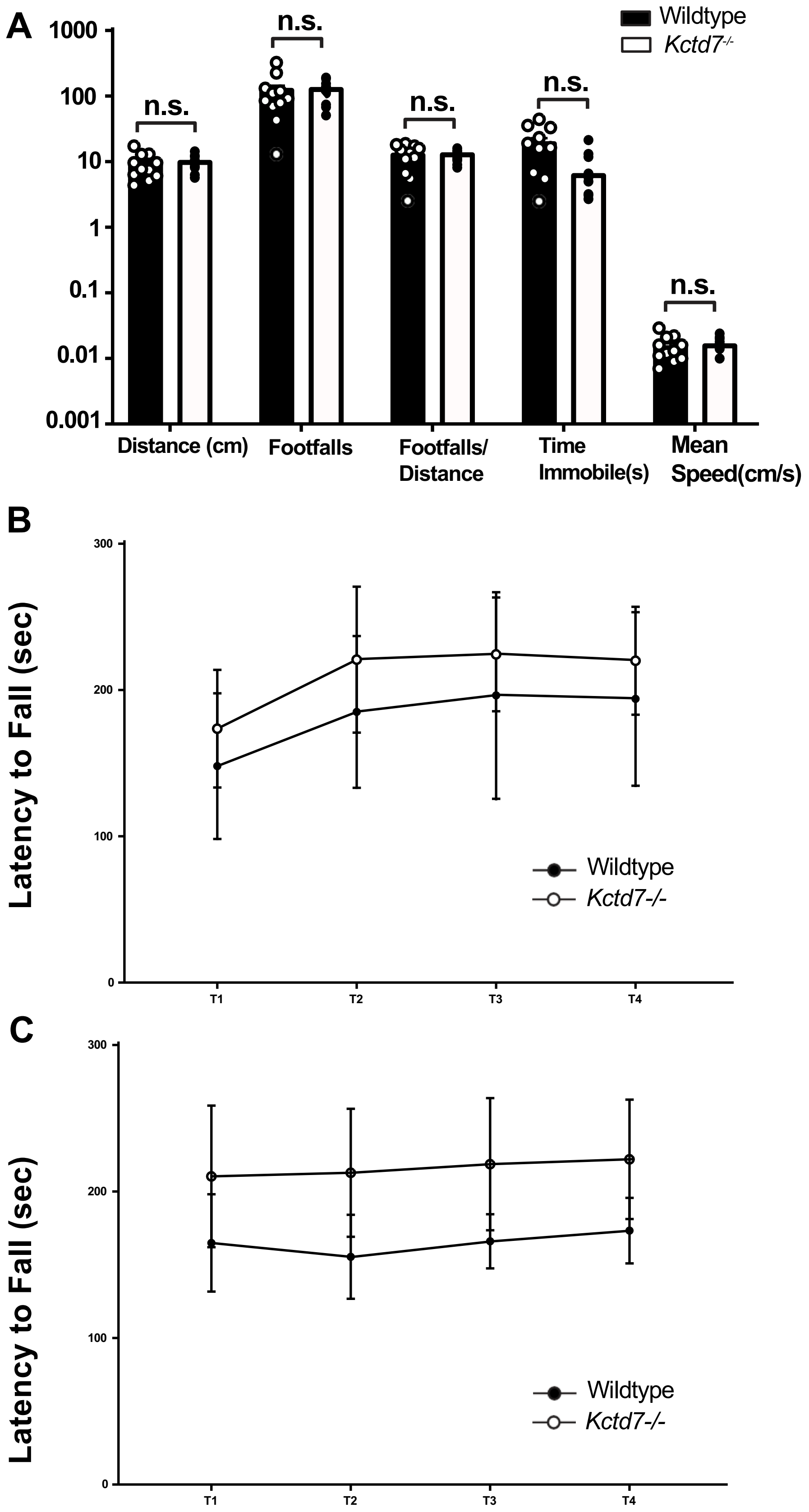


**Fig. S2**. ***Kctd7***$^{-/-}$ **mice do not show marked motor function deficits at 2 and 5 months of age.** (**A-B**). The parallel rod footslip and the accelerating rotarod test were performed on 2 month wildtype (*n = 11* and *n = 8*, respectively) and *Kctd7*$^{-/-}$ (*n = 12* and *n = 9*, respectively) mice. A footslip is detected when a paw touches a metal plate below the parallel rod floor. *Kctd7*$^{-/-}$ mice showed largely normal motor function as measured by this behavioral assay, with a small but insignificant decrease in the immobile time (**A**). Similarly, no significant deficits were noted in the latency to fall time for 2 month *Kctd7*$^{-/-}$ animals (**B**). (**C**) Accelerating rotarod analysis was also performed on 5- month-old mice wild-type (*n = 5*) and *Kctd7*$^{-/-}$ mice (*n = 8*). T values represent the standardized increasing acceleration speed. No significant defects were noted in the latency to fall time for 5 month *Kctd7*$^{-/-}$ animals. Data are represented as the mean ± the s.e.m. *** *P < 0.001*, ** *P< 0.01, * P < 0.05, 2-way ANOVA* test for significance.

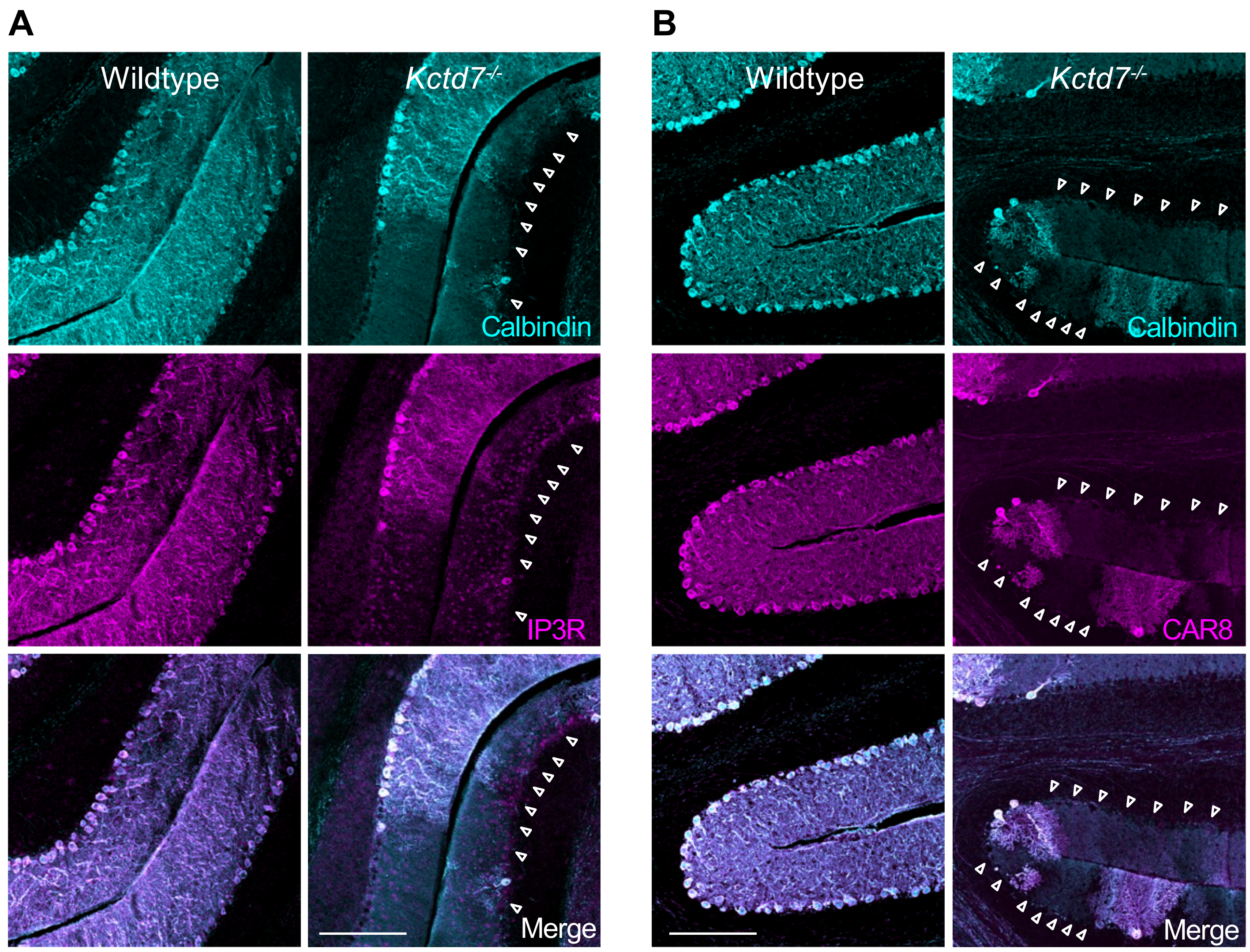


**Fig. S3. Labeling with additional Purkinje cell markers confirms cell loss in *Kctd7*$^{-/-}$ mice.** The presence and location of IP3R-positive and CAR8-positive Purkinje neurons were assayed by immunohistochemistry analysis in adult 2-month-old mice. These antibodies provide a Calbindin-independent method to visualize Purkinje neurons. In wild-type animals Purkinje neurons form a single and uniform layer present in each cerebellar lobule. In *Kctd7*$^{-/-}$ mice, clear loss of Purkinje neurons is apparent in as indicated by large gaps in the IP3R- and CAR8-positive layer (unfilled arrows). Scale bars = 200 µm.

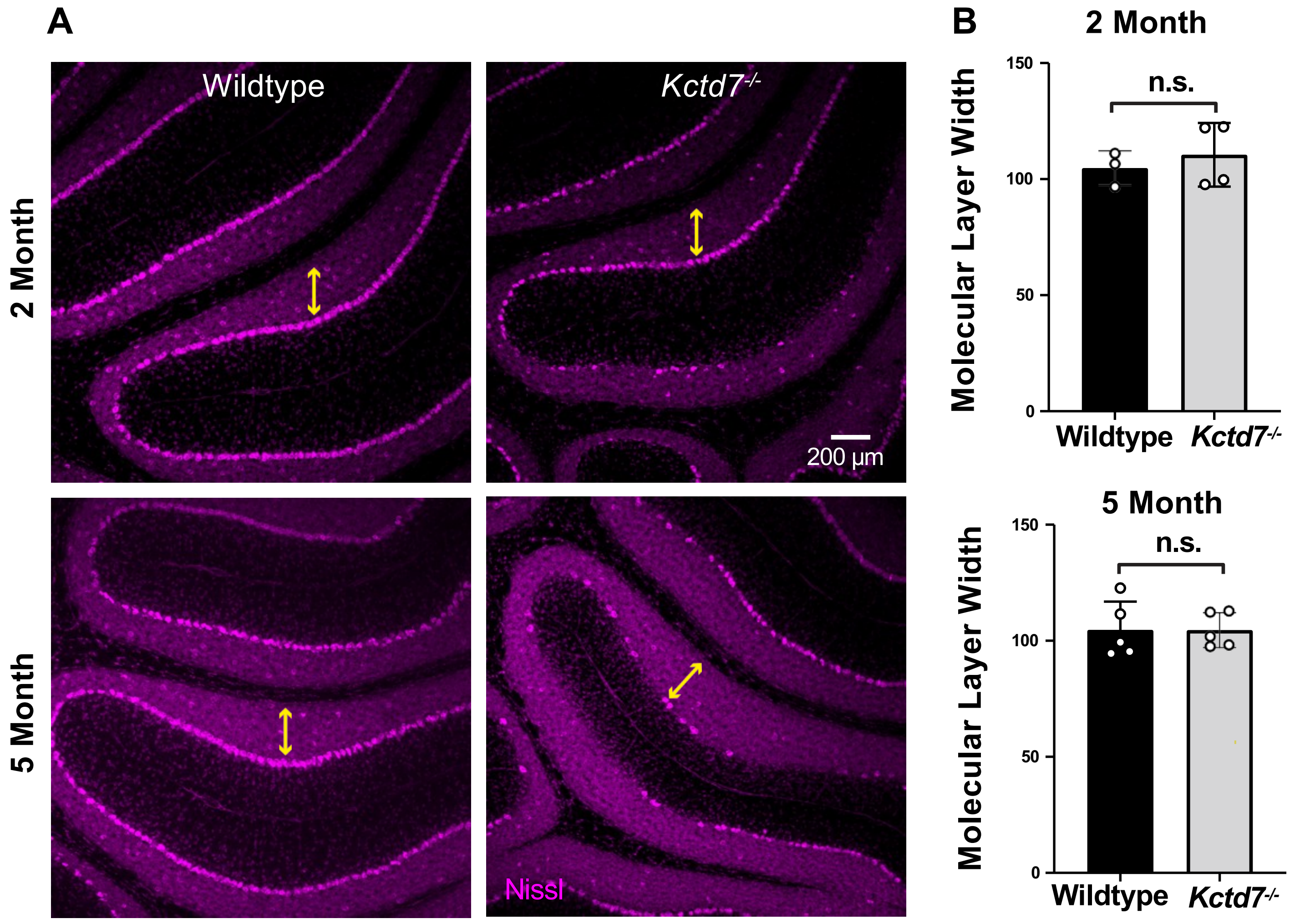


**Fig. S4. Cerebellar granular layer thickness does not change in the absence of Kctd7.** (**A-B**) The thickness of the granular layer was visualized (**A**) and measured (**B**) following Nissl staining in 2 and 5 month wild-type control (2-month *n* = *3*, 5-month *n* = *5*) and *Kctd7*$^{-/-}$ mice (2-month *n* = *4* and 5-month *n* = *5*) as a readout of cerebellar granule neuron number, size, and density. The molecular layer thickness was quantified from merged images (1272 µm x 1272 µm) using the average of 10 length measurements per lobe per animal at regions near the vermis. Kctd7-dependant alterations were not observed in the granular layer thickness. Scale bars = 200 µm Data are represented as the mean ± the s.e.m, *** *P < 0.001*, ** *P < 0.01, * P < 0.05, u*npaired t-test for significance (n.s. = not significant).

Disease Models & Mechanisms • Supplementary information

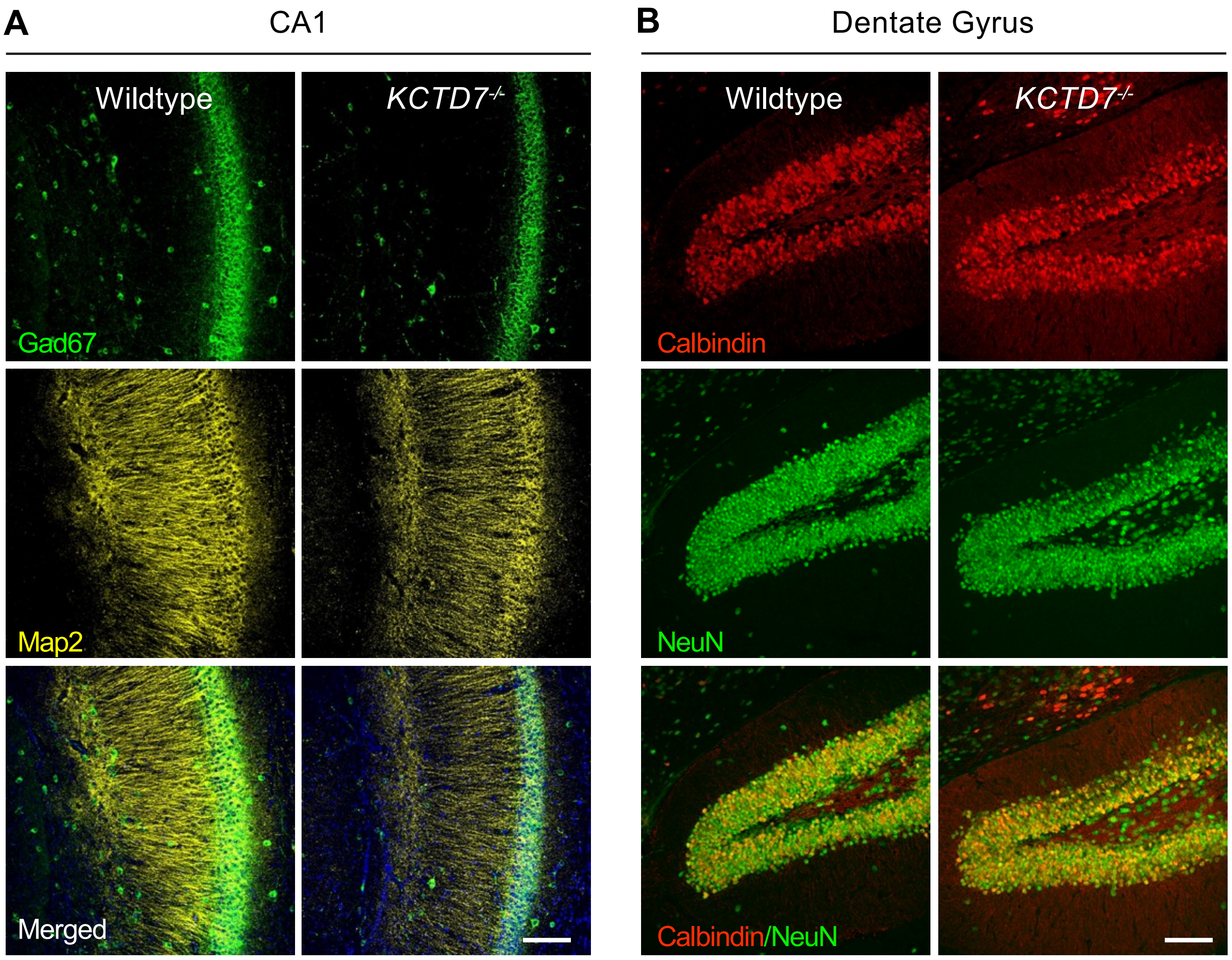


**Fig. S5**. **Hippocampal neurons appear largely normal in *Kctd7*$^{-/-}$ mice.** (**A-B**) The relative distribution and density of dentate gyrus granule cells, hilar interneurons, and CA1 pyramidal cells were examined in 2-month-old wild-type and *Kctd7*$^{-/-}$ animals by costaining for cell-type-specific markers. Grossly normal distribution of inhibitory GABAergic cells (Gad67) and all neurons (Map2) was observed in the CA1 region (**A**) of *Kctd7*$^{-/-}$ animals relative to wild-type controls. Similarly, staining for calbindin positive neuron subsets (calbindin) and all neurons (NeuN) in the dentate gyrus (**B**) did not show apparent defects in *Kctd7*$^{-/-}$ animals. Scale bar = 100 μm.

Disease Models & Mechanisms • Supplementary information

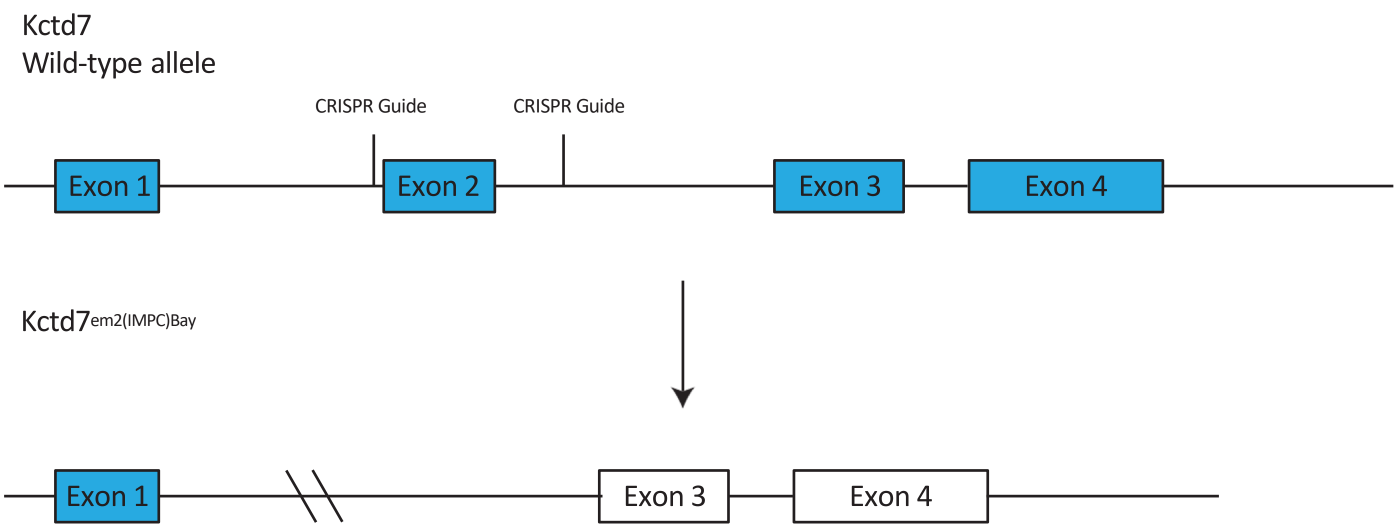


**Fig. S6. Allele schematic of $Kctd7^{-/-}$ line generation.** This mouse line was provided by the International Mouse Phenotyping Consortium ($Kctd7^{em2(IMPC)Bay}$). The allele used in this study results in the deletion of Exon2, generating a truncated protein fragment that undergoes nonsense mediated decay.

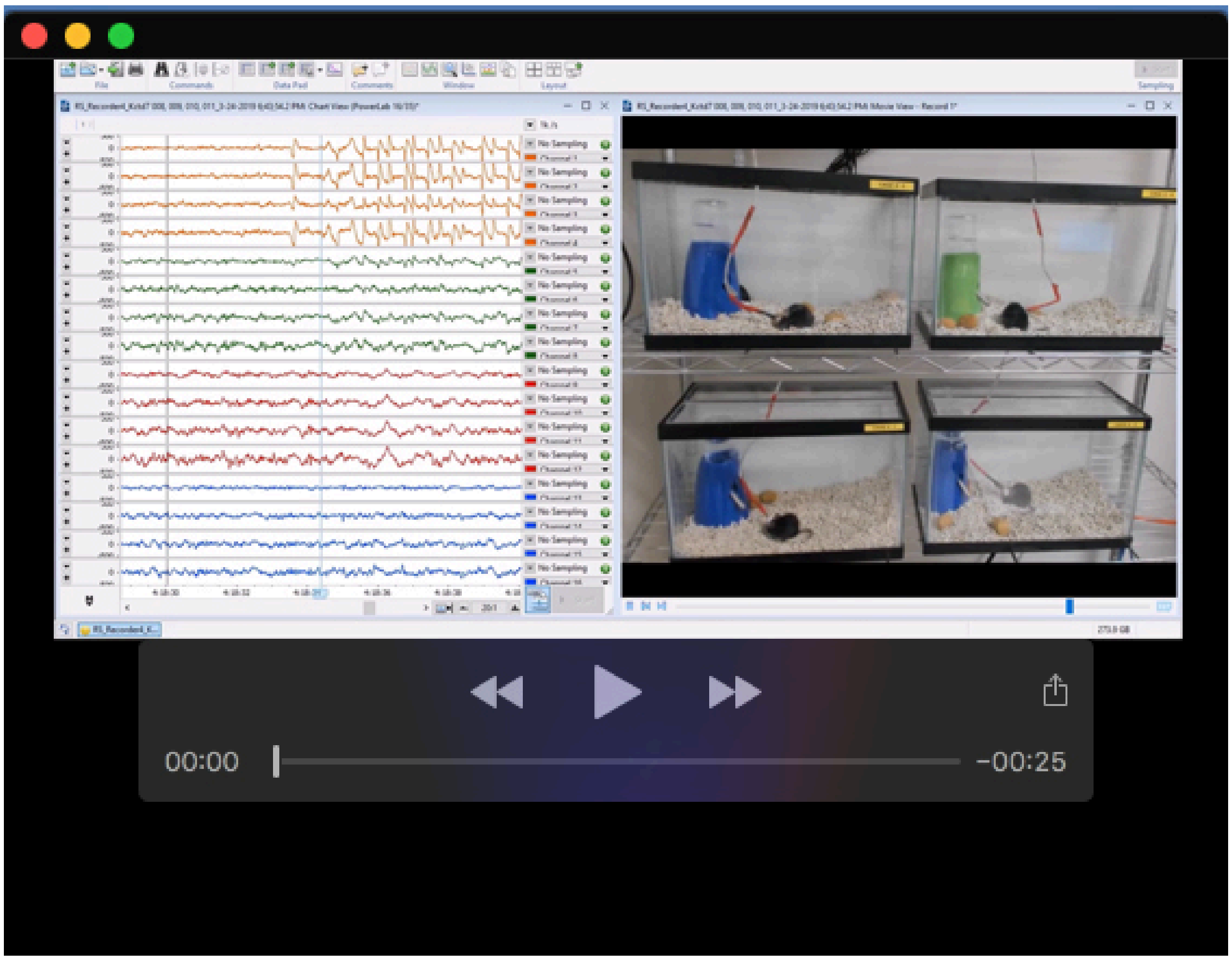


**Movie 1. Epileptiform activity in Kctd7-deficient mice**. Representative videos of EEG recording in wild-type control and *Kctd7*$^{-/-}$ animals. Among these mice, behavioral seizures were noted, consisting of high-frequency runs of spike discharges with a myoclonic head drop, followed in some instances by repetitive grooming movements of the forelimbs.
